# Accuracy of Unstimulated Basal Serum Thyroglobulin Levels in Assessing the Completeness of Thyroidectomy

**DOI:** 10.14740/jocmr1873w

**Published:** 2014-07-28

**Authors:** Emin Gurleyik, Sami Dogan

**Affiliations:** a Department of Surgery, Faculty of Medicine, Duzce University, Duzce, Turkey

**Keywords:** Thyroid, Benign disorders, Surgery, Thyroglobulin, Nuclear scan

## Abstract

**Background:**

Complete excision is important for proper surgical treatment of thyroid disorders. Functional thyroid tissue can be identified based on the level of serum thyroglobulin (Tg), which is produced only by the thyroid follicular cells, and nuclear scan.

**Methods:**

Serum thyroid stimulating hormone (TSH), free thyroxin (FT4), basal (unstimulated) Tg and anti-Tg antibody (anti-Tg ab) were measured at the sixth postoperative month in 100 patients with benign thyroid disorders treated by total thyroidectomy. Thyroid nuclear scan was obtained to identify functional remnant of the thyroid gland. The sensitivity, specificity, accuracy, positive predictive value (PPV) and negative predictive value (NPV) of the Tg levels in assessing thyroid remnant were calculated.

**Results:**

Positive scan showed thyroid remnant in 23 patients, among whom 16 were Tg positive (true positive) and seven were Tg negative (< 0.5 ng/mL) (false negative). In these patients, the nuclear scan revealed pyramidal lobe remnants. In 77 patients with negative scan, the Tg levels were also negative (true negative), and the PPV, NPV, sensitivity, specificity and accuracy of the Tg levels were 100%, 92%, 70%, 100% and 93%, respectively.

**Conclusions:**

The positive basal Tg (> 0.5 ng/mL) level accurately indicated the functional thyroid remnant after total thyroidectomy. The negative Tg (< 0.5 ng/mL) level supported complete excision of the thyroid gland. The surgical completeness of total thyroidectomy was accurately evaluated based on the serum Tg levels. Therefore, serum Tg levels should be measured in postoperative follow-up to determine the completeness of total thyroidectomy.

## Introduction

Total thyroidectomy is the surgical procedure of choice for majority of patients with thyroid gland disorders. The total removal of the gland requires complete excision of all the thyroidal tissue. The completeness of thyroidectomy has great surgical importance, mainly owing to the necessity for providing definitive treatment of both benign and malignant diseases, as well as preventing recurrence and secondary surgery. In addition, the completeness of thyroidectomy is important for sensitive and appropriate follow-up of malignant cases with biochemical analyses, nuclear medicine studies, etc. Completeness of thyroidectomy is generally assessed by examination of the presence of functional thyroidal tissue in the neck by nuclear medicine procedures. In several cases, screening with some nucleotides may not establish the presence of functional thyroidal tissue. Moreover, some focal uptakes of nucleotides in the neck do not always accurately evaluate the functional capacity and prognostic importance of the remaining tissue.

Thyroglobulin (Tg) is a large glycoprotein (MW: 660,000) stored in the thyroid follicles and produced only by the thyroid follicular cells in benign condition [1]. However, it is also produced by well-differentiated cancer cells in malignant conditions. The presence of significant amount of Tg in the serum sensitively shows the presence of functional thyroidal tissue in the body. We hypothesize that the completeness of thyroidectomy can be assessed by serum Tg levels. Hence in the present study, we aimed to determine the accuracy of serum Tg levels in assessing the completeness of thyroidectomy after surgical treatment of benign thyroid disorders.

## Patients and Methods

Between January 2010 and July 2013, a prospective study was conducted on patients with surgical thyroid diseases. Based on the following criteria, a total of 100 patients with total thyroidectomy for benign disorders were included this study: 1) The patients should have undergone total thyroidectomy. 2) Presence of benign disorders of the thyroid gland. 3) Patients with reoperative surgery were not included. 4) Total thyroidectomy should have been performed by the same surgeon to provide standard surgical technique. 5) Prescription of thyroid hormone (thyroxin; LT4) replacement postoperatively to maintain normal serum thyroid stimulating hormone (TSH) levels. 6) At the end of the sixth postoperative month, the serum TSH, free thyroxin (FT4), Tg and anti-Tg antibody (anti-Tg ab) levels were determined by biochemical analyses. 7) Total thyroidectomy cases with negative (< 2.2 IU/mL) anti-Tg ab levels were included. 8) Thyroid nuclear scan (with technetium 99 m pertechnetate) was obtained to identify the presence of functional remnant tissue of the thyroid gland.

The term “nuclear scan positive” indicated thyroid scintigraphy showing thyroid remnant in the neck. “Serum Tg negative” denoted basal (unstimulated) Tg levels < 0.5 ng/mL. “True negative” indicated Tg levels < 0.5 ng/mL in patients with negative nuclear scan image. “True positive” denoted Tg levels > 0.5 ng/mL in patients with positive nuclear scan image (Table 1). The sensitivity, specificity, accuracy, positive predictive values (PPV) and negative predictive values (NPV) of the serum Tg levels in assessing the completeness of thyroidectomy were calculated.

**Table 1:** Results of Serum Thyroglobulin (Tg) Levels and Nuclear Scan of the Thyroid Gland

	Scan negative (n = 77)	Scan positive (n = 23)	Total
Tg negative (< 0.5 ng/mL)	True negative 77 (100)*	False negative 7 (30)	84
Tg positive (> 0.5 ng/mL)	False positive 0	True positive 16 (70)	16
Total	77	23	100

*Numbers in parentheses are percentages.

## Results

**Figure 1:**
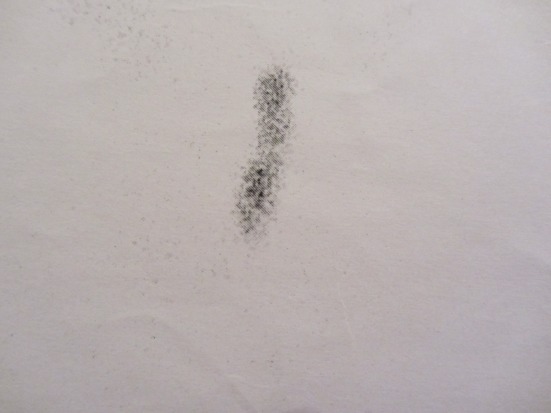
Remnant of thin pyramidal lobe. Nuclear scan and Tg positive (2.7 ng/mL); true positive.

**Figure 2:**
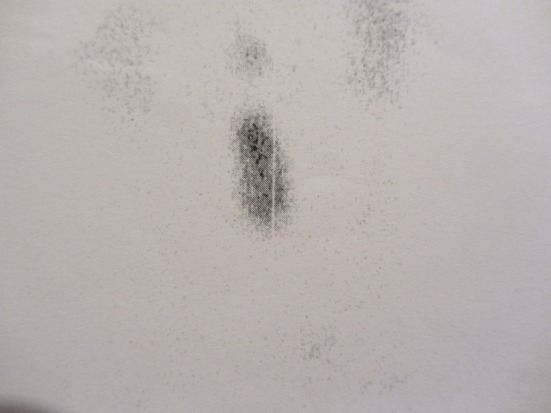
Remnant of the pyramidal lobe. Nuclear scan and Tg positive (6.45 ng/mL); true positive.

**Figure 3:**
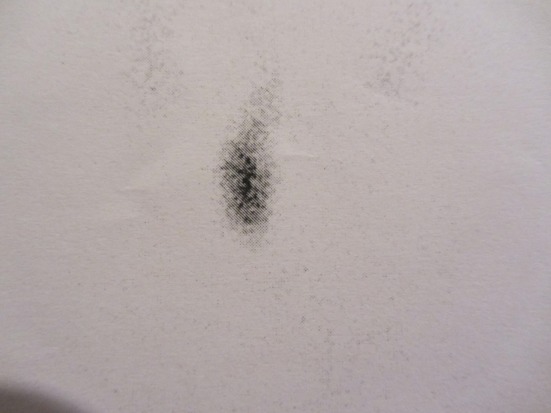
Small remnant of the upper part of the pyramidal lobe. Positive nuclear scan despite Tg negative (0.394 ng/mL); false negative.

A total of 73 patients were women with a mean age of 54 years (range, 33 - 71 years). The serum Tg levels were negative (< 0.5 ng/mL) in all the 77 patients who had no functional remnant of the thyroid gland (negative nuclear scan; true negative). Postoperative nuclear scan of the gland showed small thyroid remnants in 23 patients. The thyroid remnant was present (positive nuclear scan) in all 16 patients with positive Tg levels (true positive). However, the serum Tg levels were also negative in seven (of 23) patients with positive nuclear scan (false negative in 7% of the patients) (Table 1). In the positive nuclear scan cases, the focal uptake of the radionuclide was determined in the midline, which indicated pyramidal lobe (PL) remnant (Fig. 1-3).

The sensitivity of the serum Tg levels in assessing the completeness of thyroidectomy was 70%. Furthermore, the specificity, accuracy, PPV and NPV of the serum Tg levels were 100%, 93%, 100% and 92%, respectively.

## Discussion

Total thyroidectomy is the current surgical procedure of choice for patients with thyroid gland disorders. Complete excision of the thyroid tissue is important for definitive treatment of the disease, prevention of recurrence and secondary surgery, and sensitive follow-up of malignant cases with Tg measurement. Tg is known to be a sensitive marker of functional benign or malignant foci of the thyroid tissue. Recent studies have reported that sequential measurement of the basal serum Tg level is more effective in detecting the functional remnant of the thyroid gland after total excision [1, 2]. The 99 m Tc pertechnetate scintigraphy is a simple and feasible tool to evaluate the thyroid remnants after thyroidectomy [3]. The unstimulated baseline Tg values during TSH suppression in total thyroidectomy cases were below 0.5 ng/mL by 6 months postoperatively, and sensitive for absence of thyroid remnant [4]. In the present study, we considered thyroid scan and basal Tg levels in normal condition (normal “not suppressed” TSH levels) for the assessment of completeness of thyroidectomy 6 months postoperatively after total excision. Because thyroid gland is the only source of Tg production, serum basal Tg levels in benign cases could indicate the completeness of thyroidectomy.

In negative scan patients of the present series, the Tg levels (< 0.5 ng/mL) were also negative, whereas all the Tg-positive patients also presented positive scan images showing thyroid remnants. We found a correlation (both scan and Tg level were negative or both were positive) between the results of nuclear scan and basal Tg levels in 93% of the patients. Based on these results, it can be concluded that the positive basal Tg levels always reveal a remnant focus after total thyroidectomy. Imaging methods, especially nuclear scan, are indicated for the detection of thyroid remnant in benign cases with positive Tg level. In malignant cases, positive Tg level may indicate at least radioiodine treatment in some patients [5]. The measurement of Tg level combined with neck ultrasonography after total thyroidectomy may exclude the need for ablation in low-risk patients with Tg < 1 ng/mL [6].

Conflicting results were observed in 7% of our patients. The nuclear scan was positive despite negative basal Tg levels (false negative Tg results), and no false positive results (negative scan despite Tg positive) were noted. In a previous study, Kucukalic-Selimovic et al [7] reported 7.7% false negativity and 17% false positivity in their series. Based on the false negative Tg results observed in the present study, it can be concluded that the amount of thyroid tissue in the remnant foci determined by nuclear scan is not sufficient to produce elevated Tg levels in the basal status. Lim et al [8] observed positive scan results (thyroid bed uptake) despite negative Tg levels in 11.3% of their patients, and negative scan results despite positive Tg levels in 16.2% of their patients. Furthermore, in the follow-up period, the authors found no remarkable differences in the clinical outcome of the positive scan, Tg-negative cases [8]. Zanotti-Fregonara et al [9] speculated that in the negative-scan, Tg-positive cases, the benign sources of Tg secretion may be the foci of radio-resistant ectopic thyroid tissue. The presence or absence of thyroid remnant may be determined by nuclear applications. Unmeasurable Tg level (< 1 ng/mL) shows surgical completeness of thyroidectomy [10, 11].

It has been reported that a biochemical marker should have high PPV and NPV to accurately identify patients with thyroid remnant and reduce unnecessary diagnostic studies, respectively [1]. The PPV and NPV of the Tg level (< 0.5 ng/mL) obtained in the present study were 100% and 92%, respectively. Rosario et al [6] observed that the NPV of the Tg level (< 1 ng/mL) was 100%. Similarly, several previous studies have reported high NPV of the Tg level indicating the absence of functional foci in patients with differentiated thyroid cancer [12, 13]. In the present study, the PPV (100%) of basal Tg level in benign cases revealed the presence of functional remnant foci after thyroidectomy. These foci may be imaged by nuclear scan to establish the site of radionuclide uptake in the thyroid bed. On the other hand, high NPV of the basal Tg level may exclude diagnostic imaging studies after total thyroidectomy in patients with benign thyroid disorders. Based on previous reports and the present study, it can be concluded that in benign cases, small remnants in nuclear scan associated with negative basal Tg levels do not have clinical significance for definitive treatment and recurrence.

Incomplete thyroidectomy may create some outcome problems. The lack of definitive treatment, risk of recurrences and secondary surgeries for both benign and malignant cases, and decrease in the sensitivity of follow-up for malignant cases are the main issues secondary to thyroid remnant after thyroidectomy. In the present study, PL remnants were found in majority of our patients in the initial period of study. In the recent study period, we performed careful dissection of the anterior central compartment up to hyoid bone to excise the entire thyroid tissue. It has been reported that identification of PL is uncommon in the preoperative period and that PL is not reliably diagnosed by scintigraphic imaging. Furthermore, the percentage of PL visualization by nuclear scan is approximately 15-20% [14-16]. These are the major drawbacks in thyroid surgery and can be a cause of incomplete resection of the gland; hence special attention has to be paid to PL [15, 16]. The anterior cervical region has to be investigated very carefully during thyroidectomies. The surgeon should look for and identify PL, which should be mandatorily removed in total thyroidectomies to avoid leaving residual thyroid tissue after surgeries [15-17].

The high specificity and accuracy of serum Tg level in detecting the functional remnant of thyroid tissue after surgeries make it a promising tool for the assessment of the completeness of thyroidectomy. Serum Tg measurement is an easier and a preferred procedure for the detection of benign or malignant thyroid tissue in the follow-up after thyroidectomy. However, a minor drawback exists with respect to the sensitivity of the Tg level. Some small foci have inadequate amount of functional tissue to produce Tg in basal condition (positive scan and Tg negative), leading to false negative results of the Tg level. Previous studies have indicated that serial basal Tg measurements could elucidate doubtful gray conditions [1, 2, 7, 8, 12]. If the serial basal Tg levels remain negative in the follow-up period, it can be concluded that the clinical significance is very low and other diagnostic tools may be eliminated.

In conclusion, based on PL remnants in our cases anterior cervical region in the front of the trachea must be dissected carefully to evaluate the completeness of thyroidectomy in benign disorders of the gland. The basal serum Tg measurement is an easier tool to assess the remnant of functional tissue after thyroidectomy. The high PPV and NPV of the basal Tg levels make this glycoprotein a useful marker for the investigation of the completeness of thyroidectomy. In the postoperative period of benign cases, serum Tg level can be measured together with TSH, FT4 and anti-Tg ab levels to determine the hormone status of the patient as well as the completeness of excision.
